# *Panax ginseng* and *salvia miltiorrhiza* supplementation abolishes eccentric exercise-induced vascular stiffening: a double-blind randomized control trial

**DOI:** 10.1186/s12906-016-1139-4

**Published:** 2016-06-06

**Authors:** Hsin-Fu Lin, Kang Tung, Chun-Chung Chou, Ching-Che Lin, Jaung-Geng Lin, Hirofumi Tanaka

**Affiliations:** Department of Athletics, National Taiwan University, Taipei, Taiwan; Department of Physical Education, National Taiwan Normal University, Taipei, Taiwan; Physical Education Office, National Taipei University of Technology, Taipei, Taiwan; Brion Research Institute, Taipei, Taiwan; School of Chinese Medicine, China Medical University, Taichung City, Taiwan; Department of Kinesiology and Health Education, The University of Texas at Austin, 2109 San Jacinto Blvd, D3700, Austin, TX 78712 USA

**Keywords:** muscle damage, inflammation, arterial stiffness

## Abstract

**Background:**

Muscle damage induced by unaccustomed or eccentric exercise results in delayed onset vascular stiffening. We tested the hypothesis that a 7-day supplementation of *panax ginseng* and *salvia miltiorrhiza* prior to an acute eccentric exercise could attenuate arterial stiffening.

**Methods:**

By using a double-blind study placebo-controlled randomized design, subjects were randomly assigned to either the Chinese herb (*N* = 12) or the placebo group (*N* = 11) and performed a downhill running (eccentric exercise) trial and a control (seated rest) trial.

**Results:**

Muscle soreness increased 1–2 days after exercise similarly in both groups, whereas the herb group demonstrated a faster recovery on active range of motion. Plasma creatine kinase concentration increased significantly at 24 h in both groups but the magnitude of increase was attenuated in the herb group. Arterial stiffness as measured by carotid-femoral pulse wave velocity increased significantly at 24 h in the placebo group but such increase was absent in the herb group. Flow-mediated dilation did not change in either group. Plasma concentrations of CRP and IL-6 increased in the placebo group but no such increases were observed in the herb group. Changes in arterial stiffness induced by eccentric exercise were associated with the corresponding changes in IL-6 (*r* = 0.46, *P* < 0.05).

**Conclusions:**

A short-term Chinese herb supplementation of *panax ginseng* and *salvia miltiorrhiza* ameliorated the delayed onset vascular stiffening induced by acute downhill running exercise.

**Trial registration:**

ClinicalTrials.gov: NCT02007304. Registered Dec. 5, 2013)

## Background

Muscle damage induced by unaccustomed or eccentric exercise is associated with increases in oxidative stress, inflammatory response, and delayed onset muscle soreness [1]. The increases in circulating pro-inflammatory cytokine and C-reactive protein (CRP) are the characteristic responses induced by eccentric exercise [2, 3]. A growing body of evidence indicates that muscle damage may also exert adverse influences on vascular function a day or two days later on a similar time frame to delayed onset muscle soreness. An impairment in microcirculation [4, 5], an increase in vascular resistance [6], and a reduction in vascular reactivity [7, 8] have been observed after acute eccentric exercise. We [9] and others [10] have demonstrated that acute eccentric exercise induced significant increases in central arterial stiffness and arterial stiffening after eccentric exercise was associated with indicators of muscle damage [9].

In Chinese Medicine, Ginseng is one of the most commonly used herbs in over thousands of years [11]. Ginsenosides, the major compounds of ginseng, and its metabolites are considered to exert protective effects on the vasculature, acting as a free radical scavenger [12] and increasing nitric oxide production and antioxidant effects [13]. There have been a number of animal studies demonstrating that supplementation with either Asian ginseng (*panax ginseng C. A. Meyer*) or American ginseng (*panax quinquefolium L*.) could protect against eccentric or strenuous exercise-induced muscle damage by attenuating CK release [14, 15] and inflammatory responses [16]. Danshen (*salvia miltiorrhiza*) is another widely used Chinese medicinal herb with diverse pharmacological properties to improve circulation and blood stasis [17], including dilating coronary arteries, increasing blood flow, and scavenging free radicals in ischemic diseases [18]. Indeed danshen has been prescribed to treat angina pectoris, hyperlipidemia, acute ischemic stroke [19], and coronary heart disease [20]. The major compounds of *danshen,* Tanshinone IIA and salvianolical acid B, have been shown to suppress vasoconstrictor endothelin-1 production [21] and reduce the expression of vascular adhesion molecules in vitro [22, 23]. *Panax ginseng* and *danshen* are often mixed in herb formulas in Chinese medicine, which is characterized by adapting several types of herbs or minerals as a combination of multiple components that could synergistically attack different pathological targets [24]. *Panax ginseng* and *danshen* has been practiced as a formula to treat cardiovascular disease in Chinese Medicine [24, 25]; however, scientific evidence to support its use is still lacking. In this study we tested the hypothesis that the supplementation with a combination of *panax ginseng* and *danshen* could exert protective effects on the vasculature following eccentric exercise.

## Methods

### Participants

A total of 24 apparently healthy young male adults surrounding by National Taiwan University community were recruited. Exclusions from the study participation were due to: (1) obesity (BMI >30 kg/m^2^); (2) smoking within past six months; (3) hypertension (high blood pressure >140/90 mmHg); (4) personal history of diabetes (fasting blood glucose >126 mg/dL), history of heart disease or other cardiovascular problems; (5) orthopedic injury that may prevent him or her from completing the exercise; or (6) the use of over-the-counter supplements or vitamins. Subjects must have been sedentary or recreational active, but not been participating in any type of resistance or endurance training. All subjects gave their inform written consent prior to study participation and procedures were reviewed and approved by Institutional Review Board of National Taiwan University Hospital. This study is listed in ClinicalTrial.gov (NCT02007304).

### Experimental design

Subjects were randomly assigned into either the Chinese herb supplement or the placebo group after pre-screening and familiarization. In each group, subjects underwent two familiarization sessions followed by a pre-testing session that consists of the measurements of aerobic power and body composition. Eccentric exercise trials took place following a 7-day supplementation. Subjects were asked to keep their regular diet and sedentary lifestyle throughout the testing sessions.

### Aerobic power

Individual peak aerobic power (VO_2_peak) was determined using standard American College of Sports Medicine protocol. After a 5-min warm-up on the treadmill, subjects walked or ran while treadmill slope was increased 1 % every minute until the subjects could not continue the test. A mouthpiece and heart rate monitor were worn to collect expired air and assess heart rate throughout the test. VO_2_peak was used to set the exercise intensity during the eccentric exercise.

### Body composition

Percent body fat was measured noninvasively by using a bioimpedance analyzer Inbody 2.0 (Biospace Co. Ltd., Seoul, South Korea). To avoid the hydration effects, the test was performed in the morning when subjects were fasted.

### Supplement administration

Following the pre-testing sessions, subjects were asked to take a total of 7 capsules of either Chinese herb or placebo per day for seven days. Herb supplement was prepared in capsules consisting of 250 mg of *panax ginseng* and 250 mg *salvia miltiorrhiza* via the water-extraction method, whereas placebo capsules contained microcrystalline cellulose. According to the pharmacopoeia of the People’s Republic of China [26], the use of 1–3 g danshen extract per day is recommended [27]. A dosage of 1.75 g per day (~60 % of the maximal suggested dosage) was chosen for danshen in order to avoid unexpected adverse event and to take consideration that this was a combination herb therapy. To the best of our knowledge, a combination of panax ginseng and salvia miltiorrhiza together has not been investigated on humans in the literature [24]. Accordingly, we decided to adopt the 1:1 ratio (i.e., the same dosage) of panax ginseng and salvia miltiorrhiza as the supplementation. Similar to danshen, this particular dosage of panax ginseng has been shown to be effective. Hence, the total daily supplementation dosage was 3.5 g together.

**Fig. 1:**
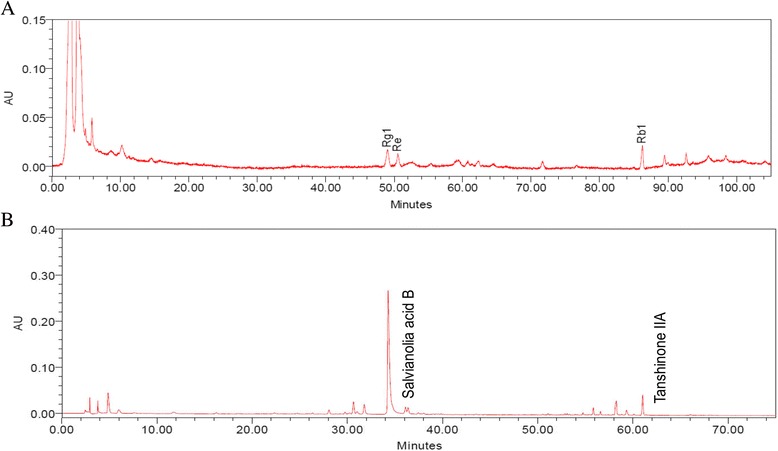
High-performance thin-layer chromatography fingerprints of Panax ginseng (**a**) and Salvia miltiorrhiza (**b**)

Both Chinese herb and placebo capsules were identical in appearance and stored in identical bottles with labeled numbers generated by a study-independent researcher. All supplement products were prepared by the Brion Research Institute, Sun Ten Pharmaceutical Co. Analyses of ginsenosides of *panax ginseng* as well as Salvianolic acid B and Tanshinone IIA in Radix *salvia miltiorrhiza* were performed by using high-performance liquid chromatography-electrospray mass (HPLC-MS) spectrometry method as previously described [26, 28]. These chromatographic quantification results of active compounds in herb supplement are shown in Table 1 and Fig. 1.

**Table 1:** Quantitative analyses of major compounds of the herb supplement used in the present study

	Compound	Content (mg/g)
*Panax ginseng*	Rb1	2.24
	Re	1.13
	Rg1	1.47
*Salvia miltiorrhiza*	Salvianolia acid B	28.2
	Tanshinone IIA	0.6

### Exercise protocol

Subjects were instructed to fast at least 8 h and refrain from any strenuous exercise for at least 72 h before the test. Experimental trial consisted of baseline measurements, downhill running (eccentric exercise) or seated rest (control), and measurements during the recovery period. In order to eliminate diurnal variation of inflammatory response to eccentric exercise, participants were asked to perform both eccentric exercise and control trials at the same time of day. Subjects warmed up on treadmill on a level grade at the speed that could elicit 75 % of pre-determined individual VO_2_peak [29] for 5 min. Each subject performed downhill running exercise on treadmill with the same speed at −10° of slope for 30 min. Similar protocols have been successfully used elsewhere to induce delayed onset muscle soreness [10, 30].

### Measurements

The measurements were made 5 times: 30 min pre, 90 min post, 24 h post, 48 h post, and 72 h post. Subjects were studied at the same time of day, during the morning hours to minimize the inconvenience of the 8-h fast and to avoid diurnal effects.

*Blood samples* were collected to determine metabolic risk factors, markers of muscle damage, inflammation, and redox state. Serum CK was used as an indicator of muscle membrane permeability or muscle damage [31]. Inflammatory markers (TNF-α, IL-6) as well as blood redox status marker, tiobarbituric acid-reactive substances (TBARS), were analyzed with the use of commercial ELISA kits. Due to the financial constraints, TNF-α, IL-6, and TBARS were measured only in the eccentric exercise condition. The inter- and intra-assay coefficients of variation were less than 10 % in all assays performed.

*Heart rate and blood pressure* were measured in the supine position. Heart rate was measured using an ECG, and blood pressure was measured using an automatic blood pressure monitor (Omron HEM907).

### Muscle soreness

Subjects were asked to rate the perception of muscle soreness using a Visual Analog Scale of 0–10 with 0 describing no soreness and 10 describing unbearable soreness immediately after a downhill running [32]. In addition, active range of motion was measured while the subjects were placed on bed in prone position with full knee extension and then moved both legs gradually to the flexion point where pain in quadriceps muscle groups was experienced. A manual goniometer was used to measure the knee angle difference from full extension to flexion point with the initiation of pain. This test was repeated three times, and the average was used for statistical analysis.

### Arterial stiffness

Arterial stiffness was measured using carotid-femoral pulse wave velocity (cfPWV), which was calculated from the traveling distance and foot-to-foot wave transit time between the two arterial recording sites in the supine position [33], and was the primary outcome measure of this study. Non-invasive pulse tonometer (SPT-301, Millar Inc. Houston TX) connected to a physiological signaling processing system (MP36, Biopac, Goleta CA) was used to detect pulse waves on the carotid and femoral arteries. The coefficient of variation for cfPWV in our laboratory were 6.2 %.

### Vascular reactivity

Flow-mediated dilatation, the secondary outcome measure, was obtained noninvasively at the brachial artery using standardized procedure [34]. Brachial artery diameter was measured using an ultrasound machine (Sonosite Ultrasound System; Bothell, WA) equipped with a high-resolution linear array transducer. A blood pressure cuff was placed on the forearm 3–5 cm distal to the antecubital fossa, and longitudinal images of the brachial artery were acquired 5–10 cm proximal to the antecubital fossa. After the acquisition of baseline measurement, the probe position was clearly marked to ensure that the image was acquired from the same location throughout the test. The blood pressure cuff was inflated to 100 mmHg above resting systolic blood pressure for 5 min by using a customized rapid inflation system. After cuff deflation, ultrasound-derived measurements of artery diameters were taken for 3 min. FMD was calculated by the following equation: (maximum diameter – baseline diameter)/baseline diameter × 100. All ultrasound images were recorded and analyzed by the same investigator who was blinded to the groups and the conditions. Our coefficient of variation of baseline diameter, maximal diameter, and FMD in our laboratory were 3.7, 4.0, and 14 %.

### Randomization and blinding

Allocation to the herb or placebo supplementation was based on a computer-generated randomization list that was prepared by a study-independent researcher. Block-randomization with a block size of four was used; the group assignment was concealed in an envelope and revealed after all data analysis was performed. During study, researchers were blinded and unaware of subjects’ allocation. Subjects were instructed not to reveal any information regarding supplement and exercise treatment during intervention.

### Statistical analyses

Descriptive statistics were used for the analyses of subject characteristics using SPSS statistical package (version 16.0; Chicago, IL). Dependent variables were analyzed within each treatment to determine the time effect (pre, 90 min, 24 h, 48 h, and 72 h post exercise) using repeated measures ANOVA. A 2-way mixed model ANOVA was used for analyses of time and treatment effects. Bonferroni post-hoc analysis was performed when significance was achieved. Associations were determined by Spearman rank correlations. To detect difference in cfPWV of 5 % at a SD of 4.5 % unit change with 80 % power (α was set at 0.05) and accounting for a 10 % attrition rate, a total of 24 subjects were to be recruited and tested.

## Results

**Fig. 2:**
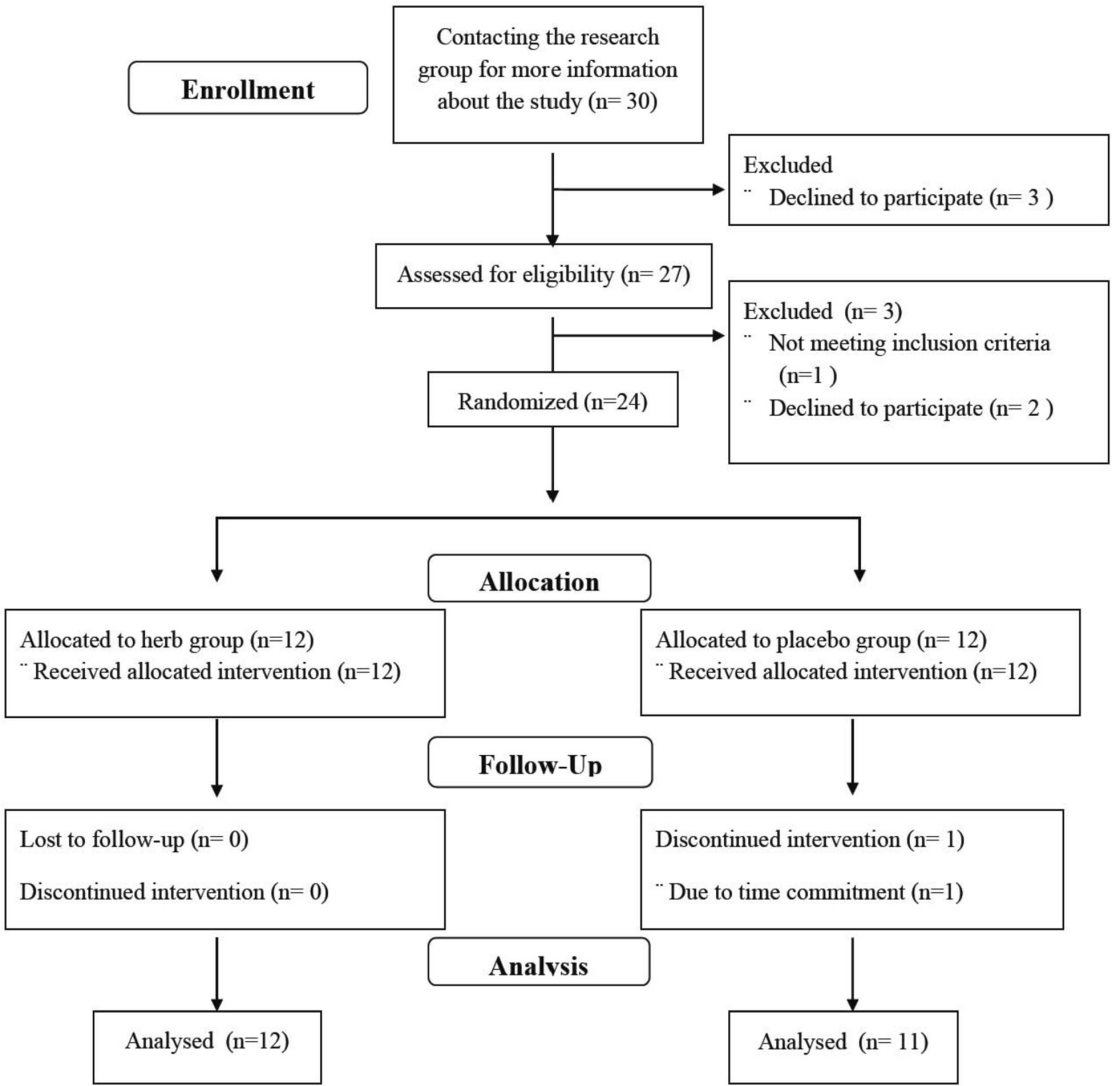
Study flow chart

After screening, twenty-four subjects were eligible and entered into the study (Fig. 2); one subject withdrew from the study during intervention due to time commitment unrelated to the study. Selected subject characteristics are presented in Table 2. All the subjects were non-obese, normolipidemic, and normotensive. There were no significant differences in body composition, lipid profile, and baseline hemodynamic parameters between the placebo and the Chinese herb groups.

**Table 2:** Selected subject characteristics

	Placebo	Herb
	(*n* = 11)	(*n* = 12)
Age, yr	24 ± 1	26 ± 5
Height, cm	173 ± 1	174 ± 3
Body mass, kg	68 ± 2	68 ± 3
BMI, kg/m^2^	23 ± 1	22 ± 1
Body fat percentage, %	19 ± 1	18 ± 2
Waist-hip ratio	0.85 ± 0.01	0.85 ± 0.01
Heart rate at rest, bpm	67 ± 3	58 ± 3
Systolic BP, mmHg	118 ± 2	112 ± 3
Diastolic BP, mmHg	66 ± 2	62 ± 2
VO_2_peak, ml/kg/min	47 ± 2	47 ± 2
HDL cholesterol, mg/dL	53 ± 2	53 ± 3
LDL cholesterol, mg/dL	97 ± 9	86 ± 5
Total cholesterol, mg/dL	180 ± 9	192 ± 8
Triglyceride, mg/dL	78 ± 9	58 ± 8
HbA1C, %	5.4 ± 0.1	5.4 ± 0.1

Values are means ± SEM

*BMI* body mass index, *BP* blood pressure, *VO*_*2*_ *peak* peak oxygen consumption, *HbA1c* glycosylated hemoglobin A1c

**Fig. 3:**
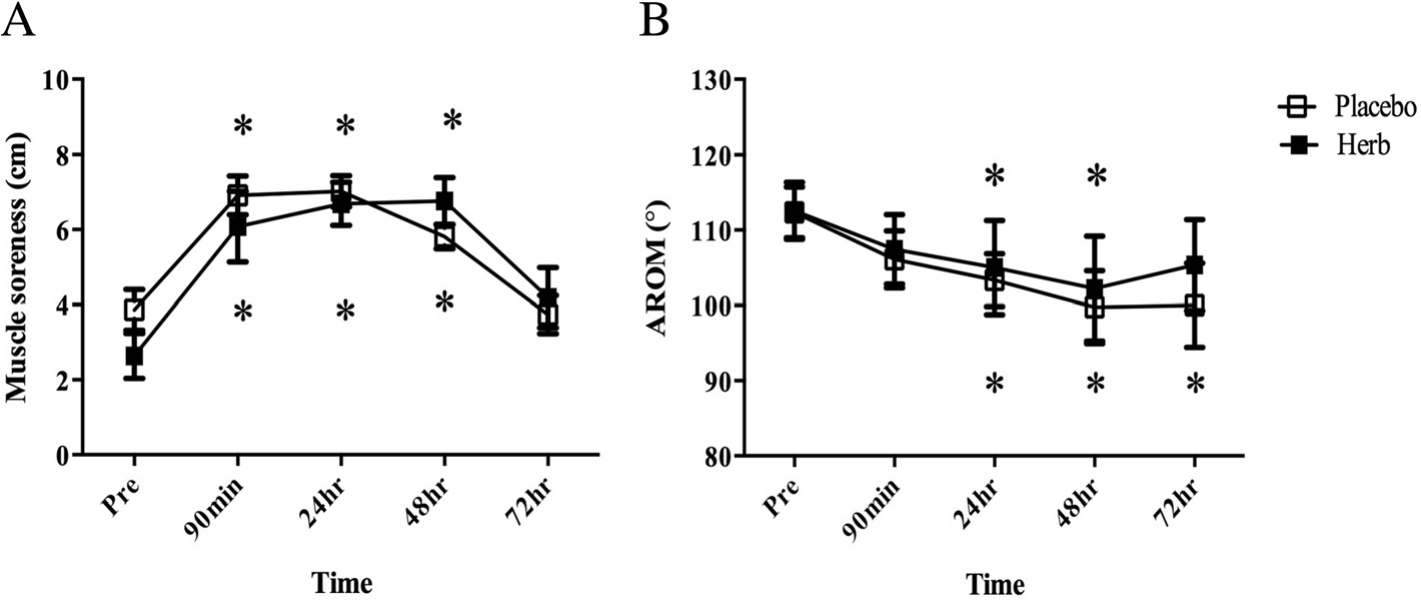
Delayed onset muscle soreness (**a**) and active range of motion (AROM) (**b**) following downhill running exercise. *P < 0.05 vs. Pre in the same condition

**Fig. 4:**
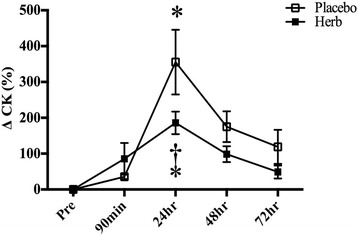
Relative changes in serum creatine kinase (CK) concentration in response to eccentric exercise sessions. *P < 0.05 vs. Pre in the same condition. †*P* < 0.05 vs. Placebo at the same time point

An acute bout of downhill running exercise increased muscle soreness significantly at 90 min, 24 and 48 h post exercise (Fig. 3). Active range of motion decreased significantly at 24 and 48 h post exercise in both groups, but remained significant at 72 h in the placebo group (Fig. 3). As shown in Fig. 4, the magnitude of the increase in plasma CK concentration was significantly greater in the placebo group than in the herb group.

**Fig. 5:**
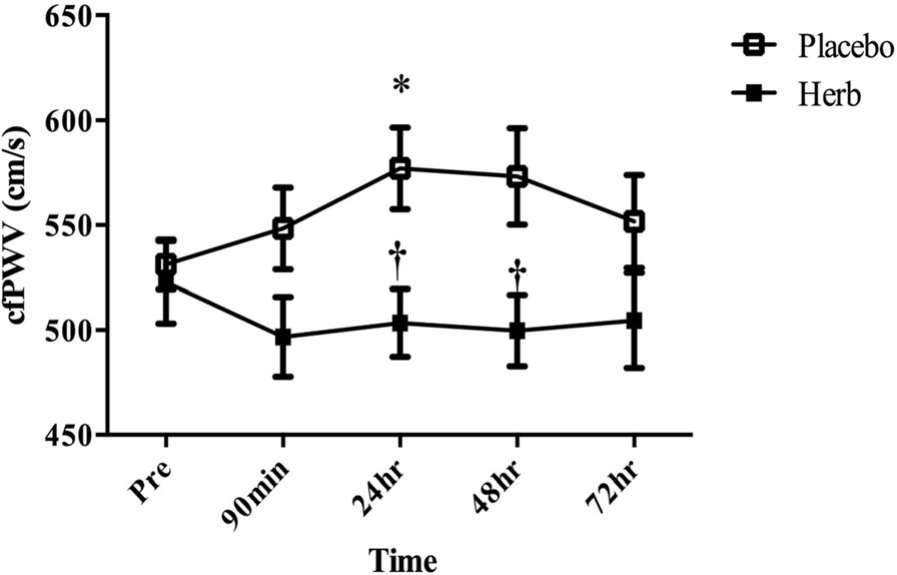
Effects of Chinese herb supplementation on carotid-femoral pulse wave velocity (cfPWV). *P < 0.05 vs. Pre. †*P* < 0.05 vs. Herb supplementation

**Fig. 6:**
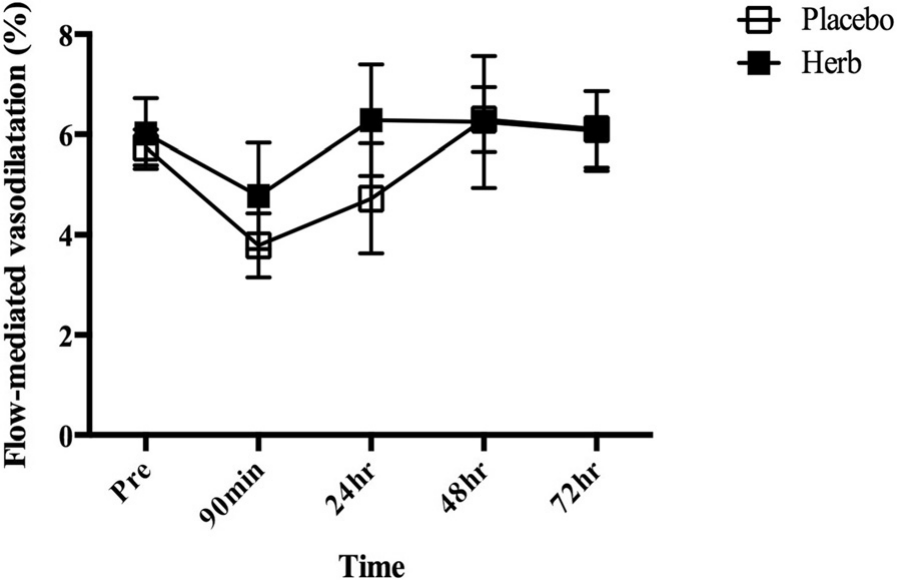
Changes in flow-mediated vasodilatation in response to the eccentric exercise

An acute bout of eccentric exercise increased cfPWV at 24 h post exercise in the placebo group (Fig. 5). No such significant increase in cfPWV was observed in the herb group. There were no changes in blood pressure in either group (Table 3). As shown in Fig. 6, there were no significant changes in FMD.

**Table 3:** Hemodynamic responses in control (seated rest) and eccentric exercise sessions

		Pre	90 min	24 h	48 h	72 h
*cfPWV,* cm/s						
Placebo	Control	530 ± 19	535 ± 17	546 ± 16	547 ± 15	513 ± 13
	Exercise	531 ± 12	548 ± 19	577 ± 20*	570 ± 24	552 ± 22
Herb	Control	482 ± 14	491 ± 17	490 ± 14	487 ± 13	505 ± 19
	Exercise	523 ± 20	497 ± 17	503 ± 16	500 ± 13	505 ± 23
*Heart rate,* bpm						
Placebo	Control	63 ± 4	58 ± 3	62 ± 3	66 ± 3	65 ± 3
	Exercise	62 ± 3	69 ± 4*	66 ± 3	63 ± 3	59 ± 5
Herb	Control	57 ± 3	54 ± 1	56 ± 3	59 ± 4	55 ± 3
	Exercise	56 ± 2	65 ± 4*	56 ± 3	55 ± 2	53 ± 3
*Systolic BP,* mmHg						
Placebo	Control	119 ± 2	116 ± 2	118 ± 2	119 ± 2	119 ± 2
	Exercise	119 ± 2	114 ± 2	120 ± 2	120 ± 3	117 ± 2
Herb	Control	114 ± 2	111 ± 2	114 ± 2	115 ± 3	115 ± 2
	Exercise	116 ± 3	114 ± 3	115 ± 2	112 ± 2	111 ± 3
*Diastolic BP,* mmHg						
Placebo	Control	66 ± 2	67 ± 1	67 ± 2	64 ± 2	67 ± 2
	Exercise	66 ± 3	65 ± 2	65 ± 3	66 ± 2	64 ± 2
Herb	Control	63 ± 3	63 ± 1	62 ± 2	62 ± 1	62 ± 1
	Exercise	63 ± 3	61 ± 2	62 ± 1	63 ± 3	60 ± 1
*Pulse pressure,* mmHg						
Placebo	Control	52 ± 2	49 ± 1	51 ± 2	55 ± 2	53 ± 2
	Exercise	53 ± 2	48 ± 2	55 ± 2	53 ± 2	53 ± 1
Herb	Control	51 ± 3	48 ± 3	52 ± 3	54 ± 3	53 ± 3
	Exercise	53 ± 3	52 ± 3	53 ± 2	49 ± 3	51 ± 3

Values are means ± SEM

*cfPWV* carotid-femoral pulse wave velocity, *BP* blood pressure

*P<0.05 vs. Pre in the same condition

Plasma CRP concentration increased significantly at 24 h post eccentric exercise in both groups (Table 4). Plasma TBARs and TNF-α concentrations did not change in either group. Plasma IL-6 concentration increased significantly at 90 min after eccentric exercise in the placebo group.

**Table 4:** Changes in muscle damage markers, inflammatory and oxidative stress markers in response to downhill running exercise in the placebo and herb group

	Pre	90 min	24 h	48 h	72 h
*Placebo*					
CRP, mg/dL					
Control	0.07 ± 0.04	0.07 ± 0.04	0.07 ± 0.03	0.06 ± 0.02	0.05 ± 0.02
Exercise	0.08 ± 0.02	0.11 ± 0.05	0.15 ± 0.05*	0.11 ± 0.05	0.10 ± 0.03
CK, U/L					
Control	94 ± 10	98 ± 8	89 ± 8	87 ± 9	90 ± 11
Exercise	93 ± 8	126 ± 13	396 ± 72^*, **^	257 ± 48^*, **^	192 ± 32*
*Herb*					
CRP, mg/dL					
Control	0.13 ± 0.05	0.14 ± 0.05	0.11 ± 0.04	0.11 ± 0.04	0.11 ± 0.03
Exercise	0.10 ± 0.03	0.09 ± 0.03	0.15 ± 0.03*	0.10 ± 0.02	0.10 ± 0.02
CK, U/L					
Control	110 ± 12	108 ± 9	119 ± 17	109 ± 11	113 ± 16
Exercise	106 ± 9	195 ± 51*	291 ± 35^*, **^	204 ± 26^*, **^	151 ± 20
TBARs, μM					
Placebo	7.1 ± 1.1	8.1 ± 1.5	7.9 ± 1.0	6.5 ± 1.0	-
Herb	7.0 ± 1.0	6.6 ± 0.8	8.1 ± 0.9	5.7 ± 0.6	-
IL-6, pg/ml					
Placebo	0.44 ± 0.1	0.69 ± 0.1*	0.32 ± 0.1	0.46 ± 0.1	-
Herb	0.50 ± 0.2	0.45 ± 0.1	0.23 ± 0.1*	0.29 ± 0.1	-
TNF-α, pg/ml					
Placebo	0.45 ± 0.14	0.28 ± 0.06	0.30 ± 0.06	0.30 ± 0.06	-
Herb	0.44 ± 0.06	0.34 ± 0.06	0.38 ± 0.08	0.34 ± 0.06	-

Values are means ± SEM. TBARs, IL-6, and TNF-α were measured only during the eccentric exercise session

*CRP* C-reactive protein, *CK* creatine kinase, *TBARs* thiobarbituric acid reactive substances, *IL-6* interleukin-6, *TNF-α* tumor necrosis factor-α

*P<0.05 vs. Pre in the same condition. **P<0.05 vs. Control or Placebo at the same time point

The associations between changes in cfPWV and changes in selected biomarkers in combined groups are shown in Table 5. Changes in IL-6 were associated with changes in cfPWV at 48 h post exercise (*r* = 0.46, *P* < 0.05). In addition, changes in TNF-α were associated with changes in cfPWV at 24 to 48 h after exercise (*r* = 0.57 ~ 0.60, *P* < 0.05).

**Table 5:** Associations between relative changes (%) in arterial stiffness and selected biomarkers

	ΔcfPWV 24 h	ΔcfPWV 48 h
ΔCK	0.05	0.08
CRP	0.17	0.21
ΔIL-6	0.11	0.46*
ΔTNF-α	0.60*	0.57*
ΔTBARs	0.09	0.36

*cfPWV* carotid-femoral pulse wave velocity, *CK* creatine kinase, *CRP* C-reactive protein, *IL-6* interlukin-6, *TNF-α* tumor necrosis factor-α, *TBARs* thiobarbituric acid reactive substances

*P<0.05

## Discussion

The major findings of this study are as follows. Seven days of herb supplementation of *panax ginseng* and *salvia miltiorrhiza* prior to downhill running exercise did not affect muscle soreness, but prevented the significant and transient increase in arterial stiffness, and facilitated the recovery of active range of motion induced by muscle damage. This "destiffening" effect was independent of blood pressure changes as arterial pressure did not change in either group. A lack of changes in arterial stiffness with the herb supplementation was in part associated with the attenuated increases in inflammatory markers. These results suggest that the Chinese herb supplementation may be an effective strategy to minimize the delayed onset vascular stiffening induced by eccentric exercise.

The presence of elevated plasma CK has been recognized a marker of increased sarcolemma permeability or muscle damage resulted from unaccustomed exercise or eccentric muscle contractions [31, 35, 36]. In the present study, an acute bout of downhill running exercise increased plasma CK concentration significantly following eccentric exercise. The increase in plasma CK concentration was greater in the placebo group at 24 h post exercise than in the herb group. Previous studies in animal models reported that North American ginseng, *panax ginseng* or *panax quinquefolus,* decreased plasma CK levels after eccentric exercise [15, 37] and preserved mitochondria integrity [14] and decreased macrophage infiltration [16] in skeletal muscle. One recent human study [38] also found that American ginseng decreased CK concentration at 72 h post exercise when compared with the placebo control. Collectively, these results suggest that Chinese herb supplementation may have reduced the amount of muscle damage induced by eccentric exercise.

In the present study, arterial stiffening effects were attenuated when the subjects were supplemented with Chinese herbs for 7 days before the unaccustomed exercise was performed. These effects were not related to changes in blood pressure in either group. To our knowledge, this is the first study to demonstrate that Chinese herb supplementation effectively prevented the adverse effects on vascular stiffening induced by eccentric exercise. Specifically, downhill running increased arterial stiffness significantly as early as 24 h after the eccentric exercise in the placebo group. In the previous study [10] including ours [9], the increase in arterial stiffness was somewhat delayed showing up at 48 h after exercise. The exact reasons for the slight differences in the time course are not known. But compared with our previous study [9] that used localized resistance exercise as an eccentric stimulus, in the present study, we used more systemic downhill running exercise that elicit greater and more robust responses [39]. Moreover, compared with the previous study that utilized moderately-trained young adults [10], the subjects in the current study were mainly sedentary adults. Current literature indicates that people who repeatedly perform eccentric muscle contractions demonstrate so-called “repeated bout effect” or adaptation in skeletal muscle that inflammatory and muscle biomarker responses were substantially lowered in the following challenge [1], suggesting that exercise training status plays a role in determining the response to this exercise challenge.

The underlying mechanisms by which muscle damage induced by eccentric exercise results in increased arterial stiffness remain unclear. However, arterial stiffening following eccentric exercise has been associated with increases in subjective muscle soreness [10], a marker of muscle damage (i.e., plasma CK) [9]. In the present study, plasma IL-6 levels increased significantly after eccentric exercise in the placebo group. Previous studies have demonstrated that the acute inflammation induced by a vaccination increased not only IL-6 and CRP concentrations [40, 41] but also arterial stiffness [42]. Collectively, these results suggest that arterial stiffening induced by muscle damage is associated with markers of muscle damage and/or systemic inflammation.

Consistent with this concept, we found that Chinese herb supplementation minimized the increases in IL-6 levels. Additionally, changes in arterial stiffness were associated with the corresponding changes in plasma IL-6 concentrations after 48 h post exercise. It is possible to speculate that the supplementation using *panax ginseng* and *salvia miltiorrhiza* reduced systemic inflammation induced by eccentric exercise and through such effect, minimized the arterial stiffening effects. Our present results using Chinese herbs as “cocktail” anti-oxidants are in accordance with a previous study [43] that supplementation with the mixture of vitamins C and E attenuated the IL-6 mRNA expression and CRP response to long-duration muscle contractions in humans.

In addition to central arterial stiffness, endothelium-dependent vascular reactivity was also measured in this study. Eccentric resistance exercise has been shown to impair FMD and linked with the increase in reactive oxygen species and a subsequent reduction in NO [7, 8]. In the present study, however, there were no significant reductions in FMD in either the placebo or the herb group. The increases in oxidative stress and inflammation have been documented in association with endothelial dysfunction [44, 45], and anti-oxidant status could play a role in modulating vascular function after eccentric exercise [46]. There were significant increases in inflammatory markers after eccentric exercise in the present study. However, FMD did not change. The discrepancy between our findings and others could be attributed to the difference in exercise mode and measured time points. Acute resistance exercise [47, 48] has been shown to reduce FMD, whereas FMD increases after an acute bout of aerobic exercise [49, 50]. The present study utilized acute downhill running exercise as a mode of eccentric exercise. FMD is also known to display biphasic response after acute exercise in that FMD decreases immediately but increases 1 to 24 h following exercise and reversed to the baseline in 48 h [46]. In the study [7] that found reductions in FMD after eccentric resistance exercise, measurements were made 45 min after exercise. We performed the measurement of FMD at 90 min post exercise. Additionally, the difference in subject characteristics may be another factor since FMD may respond differently following exercise in people of different physical fitness levels [46, 50].

There are a number of limitations in the present study that should be mentioned. First, the number of subjects studied is relatively small. Second, even though pharmacological compounds of *panax ginseng* and *salvia miltiorrhiza* were identified, it is unknown which compound of the herb supplementation was effective. Additionally, pharmacological interactions between the compounds are also unknown. Lastly, correlations between inflammatory markers and arterial stiffness were found only at selected time points. Additionally, some inflammatory markers (e.g., TNF- α) did not change with eccentric exercise. Thus, the associations should be interpreted with caution.

## Conclusions

A short-term Chinese herb supplementation incorporating *panax ginseng* and *salvia miltiorrhiza* was effective in ameliorating the delayed onset vascular stiffening induced by acute eccentric exercise, possibly via the reductions in oxidative stress and systemic inflammation.

## Abbreviations

BMI, body mass index; BP, blood pressure; cfPWV, carotid-femoral pulse wave velocity; CK, creatine kinase; CRP, C-reactive protein; HbA1c, glycosylated hemoglobin A1c; IL-6, interleukin-6; TBARs, thiobarbituric acid reactive substances; TNF-α, tumor necrosis factor-α; VO_2_peak, peak oxygen consumption
